# Microarray transcriptional profiling of Arctic *Mesorhizobium* strain N33 at low temperature provides insights into cold adaption strategies

**DOI:** 10.1186/s12864-015-1611-4

**Published:** 2015-05-15

**Authors:** Abdollah-Fardin Ghobakhlou, Anne Johnston, Linda Harris, Hani Antoun, Serge Laberge

**Affiliations:** Graduate Programs in Agri-Food Microbiology, Faculty of Agriculture and Food Sciences, Laval University, Quebec City, Quebec G1V 0A6 Canada; Eastern Cereal & Oilseed Research Centre, Agriculture and Agri-Food Canada, Ottawa, Ontario K1A 0C6 Canada; Department of Soils and Agri-Food Engineering, Laval University, Quebec City, Quebec G1V 0A6 Canada; Soils and Crops Research Development Center, Agriculture and Agri-Food Canada, Quebec City, Quebec G1V 2 J3 Canada

**Keywords:** α-proteobacteria, Genomic library, Microarray, Gene expression, Transcriptomics, Quantitative PCR, Cold adaptation, Nitrogen fixation, Arctic *Mesorhizobium*

## Abstract

**Background:**

Arctic *Mesorhizobium* strain N33 was isolated from nodules of the legume *Oxytropis arctobia* in Canada’s eastern Arctic. This symbiotic bacterium can grow at temperatures ranging from 0 to 30 °C, fix nitrogen at 10 °C, and is one of the best known cold-adapted rhizobia. Despite the economic potential of this bacterium for northern regions, the key molecular mechanisms of its cold adaptation remain poorly understood.

**Results:**

Using a microarray printed with 5760 Arctic *Mesorhizobium* genomic clones, we performed a partial transcriptome analysis of strain N33 grown under eight different temperature conditions, including both sustained and transient cold treatments, compared with cells grown at room temperature. Cells treated under constant (4 and 10 °C) low temperatures expressed a prominent number of induced genes distinct from cells treated to short-term cold-exposure (<60 min), but exhibited an intermediate expression profile when exposed to a prolonged cold exposure (240 min). The most prominent up-regulated genes encode proteins involved in metabolite transport, transcription regulation, protein turnover, oxidoreductase activity, cryoprotection (mannitol, polyamines), fatty acid metabolism, and membrane fluidity. The main categories of genes affected in N33 during cold treatment are sugar transport and protein translocation, lipid biosynthesis, and NADH oxidoreductase (quinone) activity. Some genes were significantly down-regulated and classified in secretion, energy production and conversion, amino acid transport, cell motility, cell envelope and outer membrane biogenesis functions. This might suggest growth cessation or reduction, which is an important strategy to adjust cellular function and save energy under cold stress conditions.

**Conclusion:**

Our analysis revealed a complex series of changes associated with cold exposure adaptation and constant growth at low temperatures. Moreover, it highlighted some of the strategies and different physiological states that *Mesorhizobium* strain N33 has developed to adapt to the cold environment of the Canadian high Arctic and has revealed candidate genes potentially involved in cold adaptation.

**Electronic supplementary material:**

The online version of this article (doi:10.1186/s12864-015-1611-4) contains supplementary material, which is available to authorized users.

## Background

Cold temperature is one of the most prevalent abiotic stresses on earth. Microorganisms must develop mechanisms to survive low temperature stress to occupy a cold environmental niche. Previous studies on cold adaptation have been primarily performed on mesophilic bacteria such as *E. coli*, and the main focus was on the molecular and physiological characteristics of cold shock and cold acclimation proteins (CSPs and CAPs) [1]. In addition, the role of CSPs in mesophilic bacteria has been implicated in transcription efficiency, RNA remodelling, and gene regulation at low temperature [2]. Although all psychro-mesomophilic prokaryotes react to low temperature changes by common mechanisms [3], their levels of reactions vary due to genomic diversity and specific cold adaptive features [4]. For instance, cold adaption depends on many factors, such as access to energy resources, amino acid substitution in cold-active enzymes [5, 6], effective substrate transport systems [7, 8], and diversity of occupied niches [4]. Cold temperature is perceived by various elements including membrane-associated sensors (methyl-accepting chemotaxis proteins [MCPs]) and the cytoplasmic response regulator [9, 10]. As a consequence of the action of cold sensors, a set of cold-regulated genes are activated [9], RNA/DNA conformation is altered [11], and a wide variety of genes involved in transcription, translation, and replication are affected [12, 13]. Reaction to low temperature in different cold adapted bacteria is associated with metabolic changes: increasing the membrane fluidity, integration of ice binding and antifreeze proteins (IBPs, AFPs) [14] and pigments (carotenoids) into the cell membrane [15, 16], phosphorylation of lipopolysaccharides [17], accumulation of compatible solute in cells [13, 18], and chaperone protein expression [19–21]. The compatible solutes and chaperonin proteins prevent the aggregation and denaturation of macromolecules, facilitate transcription and translation efficiency, and thus play a crucial role in low temperature adaptation [9]. A proteomic study has shown that a CSP is the low temperature trigger factor in Antarctic *Pseudoalteromonas haloplankis* and acts as the primary chaperone interacting with newly synthesized polypeptides [22]. When two chaperonin genes from the marine psychrophillic Antarctic bacterium (*Oleispira antarctica*) were transferred into *E. coli*, the recombinant cells were able to grow 38-fold faster at 10 °C and 141-fold faster at 8 °C than the parental strain [21]. It has been shown that transcript stability (half-life) is a gene regulation strategy in response to cold shock in bacteria [23]. Our metabolomics study on Arctic *Mesorhizobium* strain N33 was able to identify 110 compounds involved in central carbon metabolism, essential biosynthetic pathways, secondary metabolism, and lipids under different low temperature treatments [24]. Arctic *Mesorhizobium* sp. strain N33 [25] is a psychrotrophic N_2_-fixing symbiotic bacterium isolated from nodules of the indigenous legume *Oxytropis arctobia* from Canada’s eastern Arctic region [26]. Strain N33 is a natural streptomycin resistant mutant, and is amenable to genetic manipulation [27, 28]. Like its closely related strain N31, it has a growth range between 0 °C and 30 °C [29] and can establish an efficient N_2_-fixing symbiosis with the temperate legume sainfoin (*Onobrychis viciifolia*). This feature allows legumes to grow 40 % faster and provide 30 % higher yield compared to temperate rhizobia at low temperature on land that is cold and poor in nitrogen [30]. We hypothesized that since the *Mesorhizobium* strain N33 has the capacity to survive and grow at low temperatures, we could observe transcriptome changes in cells exposed to suboptimal temperatures. Elucidating the low temperature transcriptome may highlight molecular mechanisms of cold adaptation in this Arctic bacterium [30]. To this end, we employed a DNA microarray printed with genomic clones to investigate the partial transcriptome of strain N33 subjected to sustained suboptimal temperatures (4 °C and 10°) and transient cold treatments, compared to cells grown at 21 °C. Statistical analyses, gene annotation, and data mining revealed that microarray gene expression profiling offers new insights into how part of the genome of Arctic *Mesorhizobium* strain N33 responds to low temperature and described many molecular changes related to cold adaptation.

## Results and discussion

### Using genomic clones for transcriptome analysis under cold adaption

We prepared a genomic library containing 5760 clones from Arctic *Mesorhizobium* strain N33 (Additional file 1). The amplicons were printed on glass slides, and hybridized with labelled cDNAs using microarray technology to assess the global responses of strain N33 subjected to different hypothermic conditions. We compared gene expression during two constant low temperature conditions (GT_4_ = 4 °C and GT_10_ = 10 °C) and during transient cold stress treatments (4 °C for T_1_ = 2 min, T_2_ = 4 min, T_3_ = 8 min, T_4_ = 60 min, T_5_ = 240 min) to the reference sample at room temperature (condition T_0_, GT_21_). Microarray analysis provided reproducible results over all biological and technical replicates. After filtering, a subset of 4603 clones was generated for analysis. One-way ANOVA revealed 424 clones showing significant (p ≤ 0.005) differential expression across all microarray hybridization experiments (Additional file 2). These 424 genomic clones were sequenced from both ends using the forward (SL1) and reverse (SR2) p SMART clone vector primers. Genomic clones containing more than one gene were excluded from further consideration. The remaining clone sequences were assembled into 111 singleton clone (assembled from the SL1 and SR2 primer sequence products) and 23 contig sequences (assembled sequences from more than two overlapping genomic clone sequences), from herein referred to as genomic clones (Additional file 3).

Comparing these sequences to sequenced genomes in NCBI revealed that the closest sequenced relatives of strain N33 are *M. loti* and *M. ciceri*, exhibiting sequence identity ranging from 82 to 94 % (data not shown). This is in agreement with Laguerre et al. [25] who had evaluated the genotypic diversity of 44 rhizobial isolates originating from different geographic locations including the Canadian Arctic region and had placed strain N33 in the same 16S rRNA type as a reference *M. ciceri* strain. We also calculated that the G + C content of these sequences was 63 mol%, which is very close to the % GC of *M. loti* (62.93) and *M. ciceri* (62.56).

Self-organizing map (SOM) clustering across all treatments revealed 3 clusters of expression patterns (Additional file 3). The first cluster contained 43 genomic clones representing genes mainly up-regulated when transiently exposed to 4 °C and reduced after 240 min exposure to 4 °C or constant low temperature (GT_4_, GT_10_). The second cluster of genes represented by 28 genomic clones was mainly up-regulated at condition T_5_, and slightly at condition T_4_, as compared to the reference sample (T_0_, 21 °C). The last cluster (63 genomic clones) exhibited up-regulation during constant cold conditions GT_4_ and GT_10_. A global view of the gene expression profiles of the 134 genes in strain N33 at different hypothermic conditions revealed that the expression fold changes ranged from −11.1 to +3.71 (Additional file 3).

### Clustering of the orthologous groups of proteins (COGs)

To functionally characterize the Arctic *Mesorhizobium* partial transcriptome, the COG functional category was assigned for each gene within the 134 genomic clones (Additional file 3). The main functional categories were metabolism (37 %), cellular processes (19 %), information storage and processing (13 %), and unknown or poorly characterized function (31 %). A closer examination of the 134 genomic clones revealed 17 COG classes. COG classes C (energy production, 11.2 %), E (amino acid transport and metabolism, 11.2 %), G (carbohydrate transport, 8.2 %), R (general function prediction, 6.7 %), K (transcription, 5.2 %), and J (translation, 4.5 %) were the most-represented functional categories of genes differentially expressed during cold stress in *Mesorhizobium* strain N33 (Additional file 3). The percentage of expressed genes per category provides an overall estimate of which COG classes are most affected by cold acclimation.

### Pairwise comparisons between transcriptome profiles and annotation

**Fig. 1:**
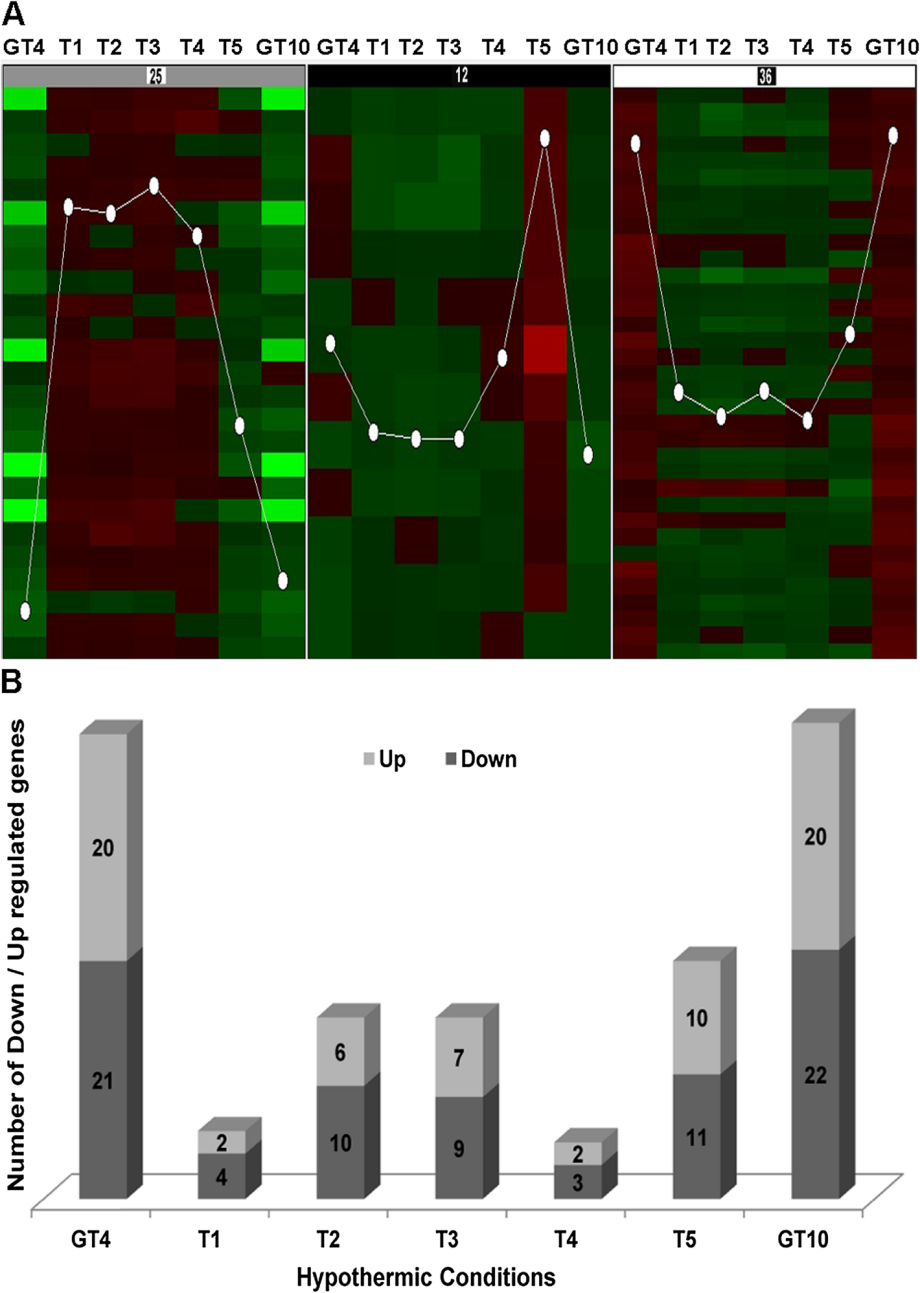
Expression profiles of 73 significantly regulated genes (p < 0.005, ≥ ± 1.5 FC) for Arctic *Mesorhizobium* strain N33. Self-organizing map clusters represent the expression patterns at different suboptimal treatments (**A**). Histograms showing numbers of significantly expressed genes under various hypothermic conditions (**B**)

We focused on the genes within the 73 genomic clones which exhibited a minimum threshold of ± 1.5 fold expression change (*p* < 0.005) between at least two treatments (Additional file 4). To group similar expression profiles, self-organizing maps (SOM) revealed 3 clusters (Fig. 1A). The first cluster contained 25 genes that were mainly up-regulated when transiently exposed to 4 °C and reduced after a 4 h exposure to 4 °C or constant low temperature (GT_4,_ GT_10_). The second cluster of 12 genes was mainly up-regulated after a 4 h cold treatment as compared to the reference sample (T_0_, 21 °C) while the last cluster (36 genes) exhibited up-regulation during constant cold conditions (GT_4_, GT_10_). The pairwise comparisons between transcriptome data indicated that more genes were differentially regulated at 4 and 10 °C, and many genes were similarly expressed under these two conditions (Fig. 1B). Likewise, a marked effect of temperature on the composition of fatty acids and water-soluble metabolites in N33 was observed in our metabolomics analysis when cells were grown under constant cold conditions at 4 °C or 10 °C [24]. We found that only a few genes were differentially up-regulated during the transient cold treatments (60 min or less; T_1_, T_2_, T_3_, and T_4_), except for condition T_5_ (4 h) which showed an intermediate response (Fig. 1B).

### qRT-PCR validation of microarray results

**Fig. 2:**
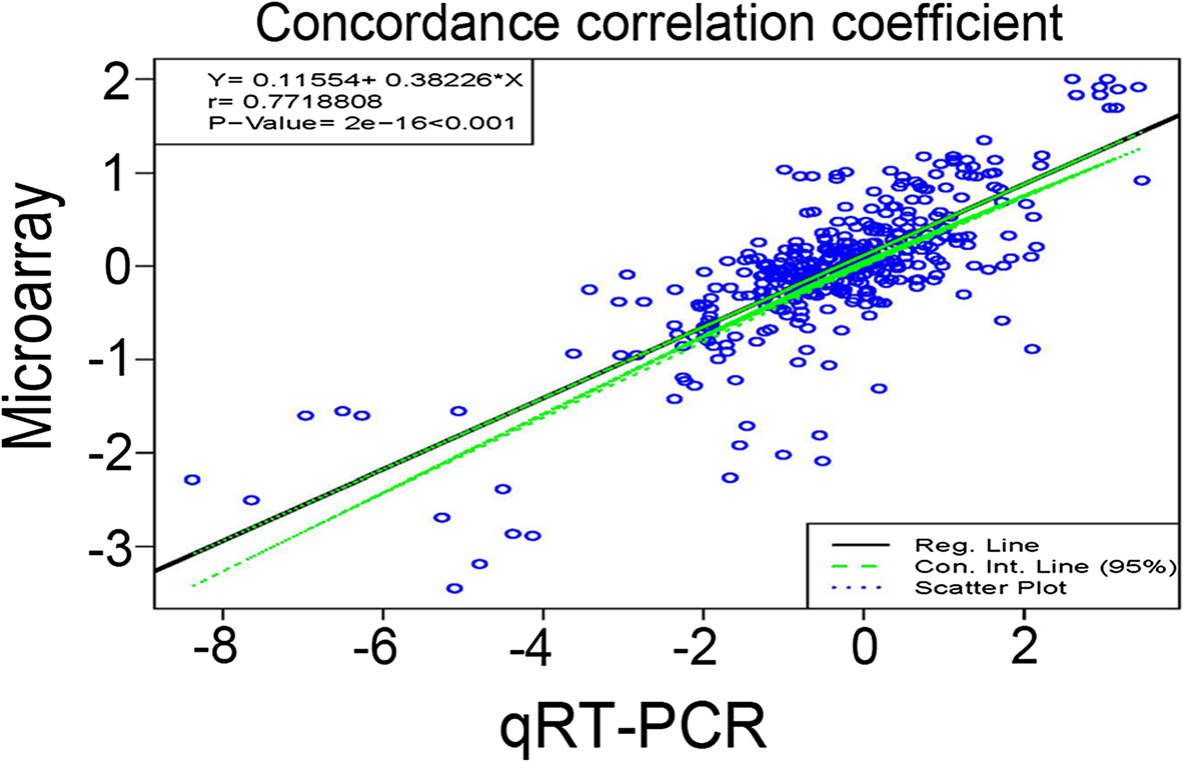
Correlation between qRT-PCR and microarray data: Correlation between the Log_2_ fold change of 54 genes (with 8 samples over 3 biological replicates) in qRT-PCR and microarray data from Arctic *Mesorhizobium* strain N33 is represented

Quantitative Real-Time PCR (qRT-PCR) was performed on the genes identified within 54 selected clones to confirm the gene expression profiles suggested by the microarray data. Based on the geNorm algorithm analysis [31], we identified six genes (Additional file 5) whose expression levels showed very little variability and represented average expression stability below 0.15 (M ≤ 15). We selected the three most stable transcripts (clones MrN33-01a03, MrN33-19d03, and MrN33-04d09) and applied them as references for qRT-PCR gene expression analysis [32]. The REST-2008 software [33] showed statistically significant (p < 0.05) differences in relative gene expression among the 54 selected transcripts. The pair-wise comparison of the Log_2_ expression ratios between 54 clones data obtained by qRT-PCR and microarray showed a consistent (|r| = 0.77, p <0.001) correlation coefficient between the two technologies [34] (Fig. 2, Additional file 5).

### KEGG and COG classification

**Fig. 3:**
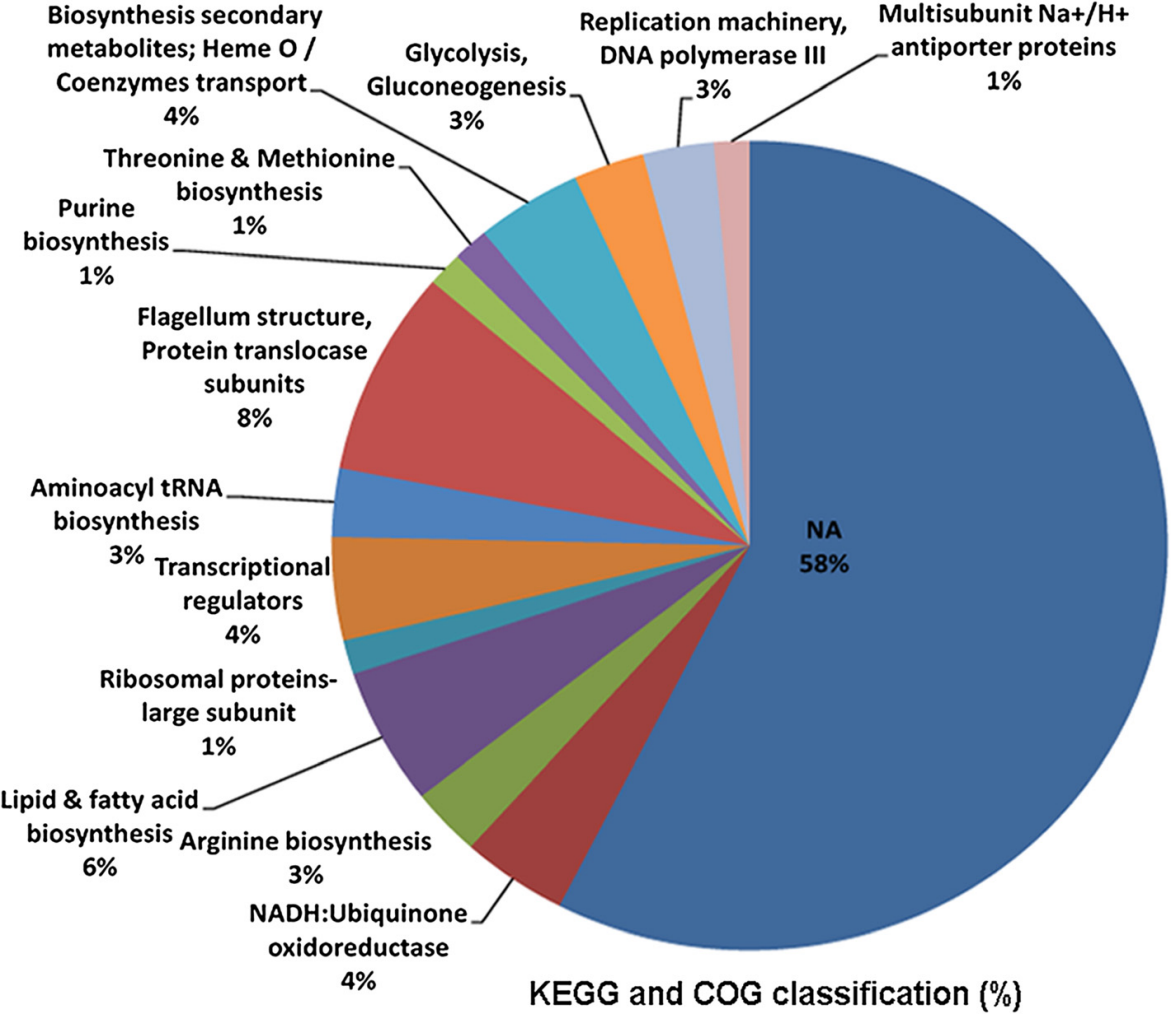
Categories of 73 significant genes assigned in KEGG and COG databases: The 73 significant (p < 0.005, ≥ ± 1.5 FC) genes were classified into 14 different categories with diverse cellular functions. NA = Not available in KEGG / COG databases

The 73 selected sequences exhibiting >1.5 fold change in expression were classified into 14 different categories using the KEGG and COG databases indicating their involvement in many different cellular functions during cold acclimation (Fig. 3; Additional file 4). Fifty eight percent of the sequences could not be assigned to any category (NA). These putative genes suggest a large knowledge gap in prokaryotic cold adaptation. Other studies have also identified many differentially expressed transcripts encoding unknown functions under sub-zero growth in *Exiguobacterium sibiricum* and *P. halocryophilus* Or1 [7, 35], which are more suitable for further investigation. The most prevalent categories include lipid and fatty acid biosynthesis, flagellum structure, protein translocation, transcriptional regulation, ribosomal proteins, replication machinery, aminoacyl-tRNA biosynthesis, secondary metabolite biosynthesis, Heme O / coenzymes transport, energy production and arginine metabolism, replication machinery and DNA polymerase III, glycolysis and gluconeogenesis.

### Signal transduction

**Fig. 4:**
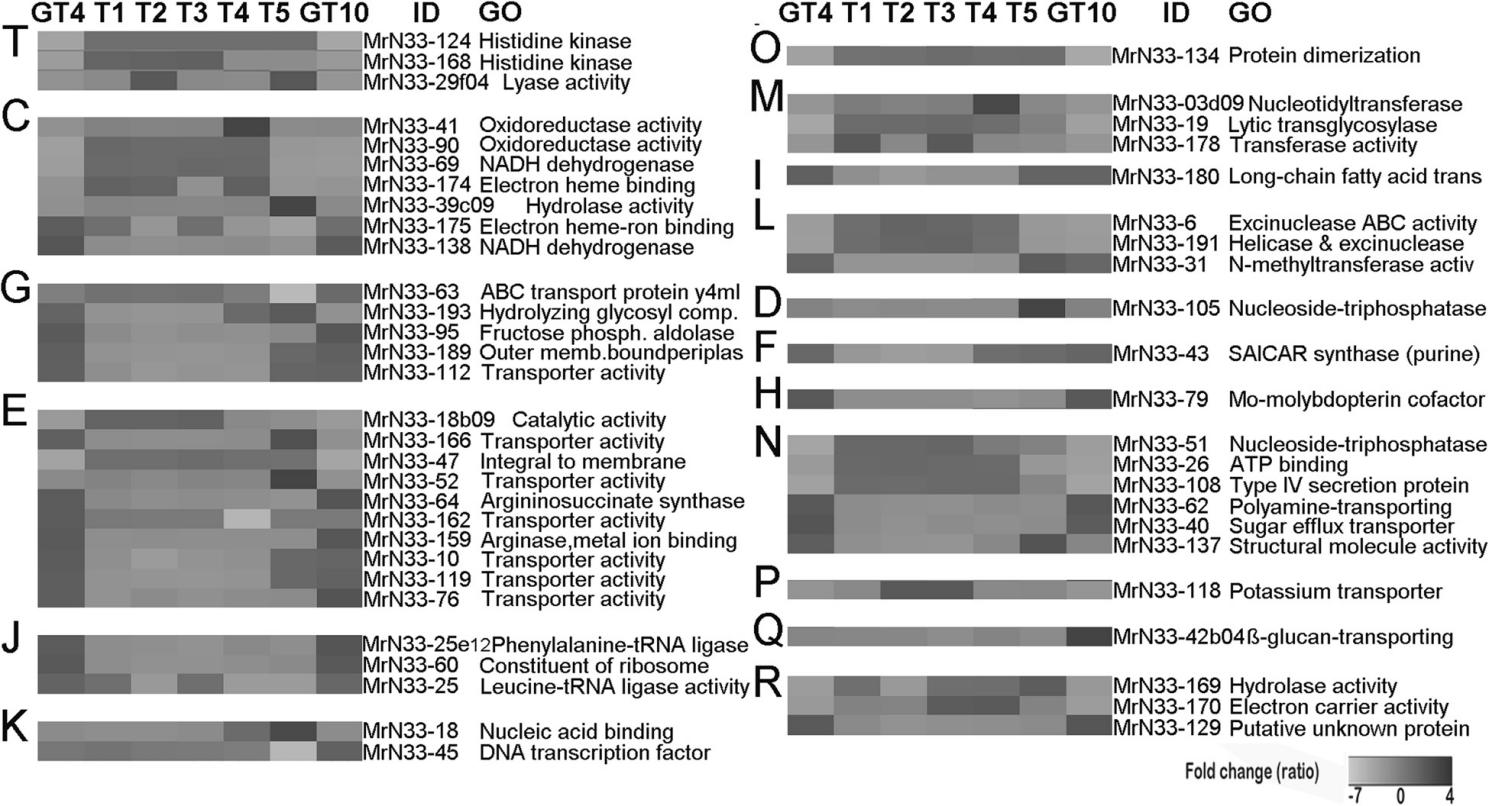
Heat maps of selected differentially expressed genes of Arctic *Mesorhizobium* strain N33. Genes were selected from 73 genomic clones/contigs (Additional file 4), grouped by clusters of orthologous group (COG) category from T to R (see Additional file 6), and described by gene ontology (GO). Comparisons between transcriptomics data sets of different cold treatment conditions (GT_4_, T_1_, T_2_, T_3_, T_4_, T_5_, and GT_10_) are shown in columns. Values are expressed as ratio fold changes (−6.67 to 3.71). The 52 selected genes were differentially expressed in at least one data set (p < 0.005, ≥ ± 1.5 FC), and the corresponding data is available in Additional file 6. Gene expression fold-change ranges from black (up regulation) to light grey (down regulation)

The survival of cells requires the ability to sense environmental conditions [36]. Unlike other bacteria [9] whose two-component signal transduction histidine kinase genes were mainly up-regulated and involved in early perception of cold stress, we did not identify any histidine kinase genes clearly upregulated during early cold stress. However, as only a partial transcriptome was profiled, there may be more potential histidine kinase genes in strain N33 that were not explored in this study. During constant cold conditions (4 and 10 °C), the expression levels of three genes (represented by MrN33-29f04, MrN33-168, and MrN33-124) were suppressed (Fig. 4, group T; Additional file 6). It has been shown that two component signal transduction systems can be involved in many functions including secretion, biofilm formation, cell differentiation and targeted transcriptional regulation [37]. Down regulation of two histidine kinase genes (MrN33-168 and MrN33-124) in N33 could be involved in the control of metabolite secretion at low temperatures since histidine kinases can act as communication interfaces in bacteria [37].

### Energy production, cryoprotectants, carbohydrate transport and metabolism

The majority of genes required for energy production that are represented on the array are down regulated at constant suboptimal conditions of 4 and 10 °C (Fig. 4, group C; Additional file 6), with the exception of the NADH dehydrogenase (MrN33-138) and the electron carrier heme-iron binding encoding gene (MrN33-175). Transcript levels of one heme-binding electron carrier gene (MrN33-174) increased after short-term cold treatment (T_1_ = 2 min, and T_4_ = 60 min). The expression levels of a putative hydrolase gene (MrN33-39c09) increased during the 240 min treatment at 4 °C (Fig. 4, group C; Additional file 6). The increased abundance of these above-mentioned transcripts may help to maintain cellular energy metabolism at low temperatures to compensate for low growth rate [38]. Despite the repression of many genes involved in energy production under cold stress conditions in *Mesorhizobium* strain N33, transcript levels encoding proteins in carbohydrate metabolism increased, possibly to compensate for nutrient limitations.

Transporter proteins in bacteria are at the primary interface with the environment. ABC transporters are a major family of membrane transport proteins that enable the acquisition and efflux of a broad range of nutrients and metabolites in cells [39, 40]. Several genes (Fig. 4, group G; Additional file 6) encoding predicted transporters (MrN33-112, MrN33-95, MrN33-63, and MrN33-189) are induced during constant growth either at 4 °C or 10 °C, some of which may be responding to the need for carbohydrate reserves under cold stress. Genomic clone MrN33-189 encodes a predicted periplasmic mannitol-binding protein (PMBP) and is induced during constant cold conditions (GT_4_ and GT_10_), and suppressed in transient cold exposure conditions; this protein may assimilate mannitol from the culture media to increase cold tolerance. In addition to a carbohydrate source, mannitol is a cryoprotectant and compatible solute which prevents the aggregation and denaturation of cellular proteins [9]. Cryo-protectants (metabolites and proteins) stabilize cytoplasmic macromolecules and phospholipid bilayers, prevent or reduce ice-crystal formation and freezing damage in bacterial cells at low temperatures [41, 42]. Metabolomics analysis of *Mesorhizobium* strain N33 has confirmed that mannitol accumulates at constant cold growth conditions (GT_4_ and GT_10_), as do other compatible solutes (isobutyrate, sarcosine, threonine and valine) [24]. A reverse genetics experiment [43] could elucidate whether mannitol is assimilated from the culture media or synthesized de novo.

### Amino acid, nucleotide transport and metabolism, translation and ribosomal biogenesis

Arctic *Mesorhizobium* strain N33 transcription profiles indicate the induction of amino acid and nucleotide transport systems likely to increase protein biosynthesis during growth at constant low temperatures. In particular, the expression levels of many genes that encode purine biosynthetic enzymes (MrN33-43), amino acid metabolic enzymes (MrN33-64, MrN33-159), and transport proteins (MrN33-76, MrN33-10, MrN33-119, MrN33-162), increased during cold treatment, indicating that individual amino acids, proteins and purines may be used as nitrogen and carbon sources, or could be used in new protein synthesis [7, 13] (Fig. 4, groups E, F; Additional file 6). In our previous metabolomics study, we determined that amino acids sarcosine, threonine, and valine levels increased during growth at 4 °C and after short term exposure to 4 °C; thus, they could play a role in conferring cold acclimation to strain N33 by acting as cryoprotectants [24]. We also have observed that some genes involved in translation and ribosomal biogenesis increased in *Mesorhizobium* N33 growing at constant suboptimal temperatures of 4 and 10 °C (Fig. 4, group J; Additional file 6).

### Defence against reactive oxygen species (ROS), protein turnover and chaperones

At low temperatures, the solubility of gases and stability of reactive oxygen species (ROS) increase and would be deleterious to cells [22]. Some psychrophillic bacteria have developed mechanisms to protect their cells against ROS for growth at low temperatures [7, 13, 22]. For instance, *Colwellia psychrerythraea* and *Desulfotalea psychrophila* each have several copies of catalase and superoxide dismutase genes to protect their cells against deleterious ROS [44]. *Planococcus halocryophilus* Or1 is able to protect against ROS by inducing five genes encoding MsrA, a methionine sulfoxide reductase involved in reversing oxidative damage to methionine [7]. In our experiment, Mo-molybdopterin cofactor (clone MrN33-79), involved in coenzyme transport and metabolism (Fig. 4, group H; Additional file 6), is induced in N33 cells grown at 4 and 10 °C. This cofactor is an essential element for diverse enzymes involved in protecting the cells against toxic compounds including ROS and increases the adaptability of psychrophilic bacteria to cope with low temperatures [22, 45]. The genome sequence analysis of the Antarctica bacterium *P. haloplanktis* TAC125 [22] indicated how the genome has adapted to protect the cells against ROS.

### Transcription factors and regulators

**Fig. 5:**
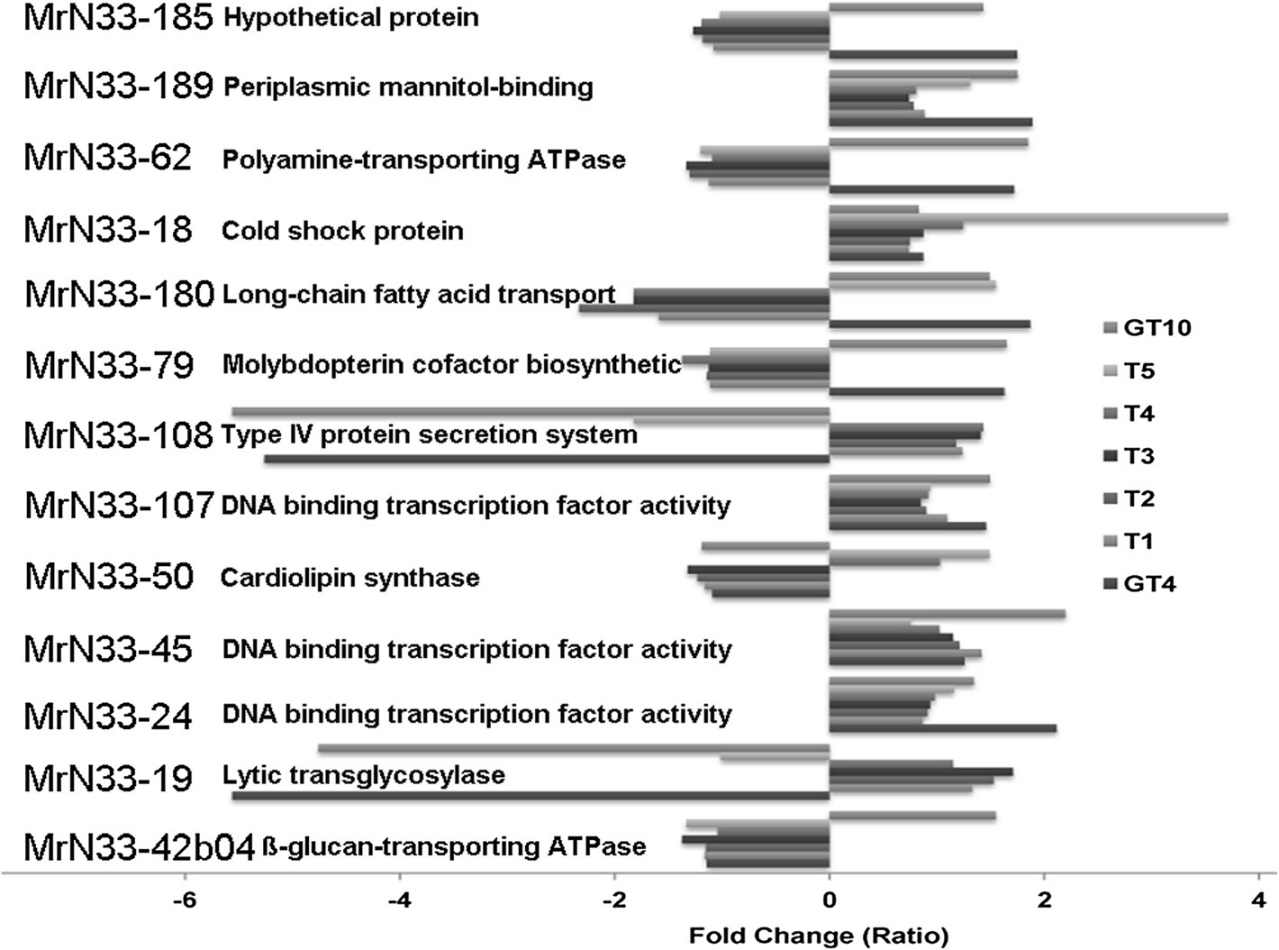
Selected genes related to cold tolerance that are up-regulated in at least one hypothermic treatment condition. These are candidate genes for further functional investigation using reverse genetics. The data for up regulated and down regulated genes are shown based on fold change ratio relative to room temperature conditions. The seven hypothermic treatment conditions (from GT_10_ to GT_4_) are depicted on the graph on the right and the name of the representative genomic clone / contig is listed on the left

Genomic contig MrN33-45 contains a DNA binding transcription factor gene that was induced at a constant cold treatment of 10 °C (Fig. 4, group K; Fig. 5; Additional file 6). The gene within clone MrN33-18, encoding a cold shock protein with nucleic acid binding activity, was suppressed under constant cold treatment (GT4, GT10) but induced after 1 h and 4 h (T_4_, T_5_) exposure to 4 °C (Fig. 4, group K; Additional files 5 and 6). Cold shock proteins act as chaperones, are involved in protein and nucleic acid folding, facilitate mRNA unwinding, translation initiation, ribosome assembly, RNA metabolism and degradation at low temperature in cold adapted bacteria [20, 21, 44, 46]. It has been reported that an excess of cspA mRNA is poisonous to the cells [47], and the protein level may remain high only up to a certain time at low temperatures [48].

### Cell envelope, outer membrane biogenesis, and lipid metabolism

The bacterial cell wall and cell membrane act as protective barriers against many environmental stresses including low temperature. The expression levels of three genes (represented by MrN33-178, MrN33-19, and MrN33-03d09) decreased during constant cold treatment of 4 °C and 10 °C and their respective proteins are predicted to possess glycosyl transferase activity, lytic transglycosylase and nucleotidyl transferase activity (Fig. 4, Group M; Additional file 6). A predicted lytic transglycosylase (MrN33-19) was induced at transient suboptimal temperatures (T_1_, Additional file 5). Lytic transglycosylase acts on peptidoglycan, is responsible for creating space within the peptidoglycan chamber for its biosynthesis and recycling, cell division, and cell wall metabolism, and is required for the assembly of the flagellum and the pilus [49]. Our results corroborate previous observations indicating that different cell envelope modifications were affected at low temperature in the gram-negative *Psychrobacter arcticus* [50]. The down regulation of those three transcripts can be seen as an adjustment to low temperature as has been observed for many genes involved in energy metabolism. We observed that a gene within clone MrN33-180, which encodes a long-chain fatty acid transport protein, was significantly up regulated when cells were grown in steady cold at 4 °C or 10 °C and in response to 4 h exposure to 4 °C (T_5_), and suppressed at transient suboptimal conditions (Fig. 4, group I; Additional file 6). Principal components analysis (PCA) of the metabolomic fatty acid profiles from the four lipid classes [24] revealed that N33 cells grown at suboptimal low temperatures (4 °C and 10 °C) are clustered into a distinct group from the cells exposed to 4 °C for 2 to 240 min, thereby suggesting a significant association between gene expression and metabolite stress response at low temperatures. These findings strongly suggest that lipid metabolism and fatty acid changes in strain N33 might be essential for the adaptation to temperature downshifts. Conversion of saturated to unsaturated fatty acids and branched fatty acids in the cell membrane and the biosynthesis of new phospholipid head groups provide the necessary cell membrane fluidity to withstand low temperatures [7, 13, 51].

### DNA Replication, recombination and repair

The gene represented by clone MrN33-31, encoding a methyltransferase, is significantly up-regulated after 240 min (condition T_5_) of cold acclimation and suppressed during transient cold acclimation. Genes within contig MrN33-191, encoding a DEAD-box protein which may act as a RNA helicase, and MrN33-6, encoding an excinuclease, were suppressed at 4 and 10 °C, but were shown to increase during transient cold treatments after 4 min (T_2_) and 8 min (T_3_) (Fig. 4, group L; Additional files 5 and 6). The role of RNA helicase has been studied in different psychrophillic microorganisms [19] in which it helps to unwind the RNA secondary structure for efficient transcription and translation at low temperature. Previous studies on psychrophillic and mesophilic prokaryotes have indicated the role and importance of exoribonuclease, chaperonin proteins, helicase (DEAD-box proteins) in nucleotide excision repair pathways, RNA degradation and cold adaptation [2, 19, 46, 52]. It was shown that RNA degradation at low temperatures helps the cells to adapt its RNA metabolism for subsequent growth at low temperatures [2]. These changes provide cold tolerance ability by facilitating many cellular functions including transcription efficiency, RNA and protein integrity, translation initiation and ribosome assembly at low temperature [19, 20].

### Cell motility, secretion and biofilm formation

Six genes involved in secretion and cell motility are regulated by low temperature treatments (Fig. 4, group N; Additional file 6). The gene within contig MrN33-137 is induced during prolonged cold acclimation (T_5_) at 4 °C and encodes a flagellin (flaA) protein involved in the flagellum structure and biogenesis pathway (Fig. 3). Clone MrN33-40 represents a gene encoding a sugar efflux transmembrane transporter and is significantly up-regulated during constant growth at 4 °C. This transporter may be involved in the production of EPS (exopolysaccharide) and biofilm formation and help to protect the cells against cold temperature [53]. In this work, we observed EPS production increase in *Mesorhizobium* strain N33 during constant growth at 4 °C. It has been shown that genes involved in EPS synthesis, such as tyrosine kinase (EpsD) and glucose-1-phosphate thymidylyltransferase, were induced at −15 °C in psychrophillic bacterium [7]. In addition, the gene within clone MrN33-62, encoding a polyamine-transporting ATPase, was induced at both 4 and 10 °C acclimation (Fig. 5). In *E.coli*, it has been shown that polyamines act as compatible solutes under cold acclimation conditions to help provide appropriate ionic conditions and promote the initiation of protein biosynthesis by the ribosome [54]. We also point out that two genes (MrN33-108, MrN33-51), which encode membrane transporter processive enzymes for secretory proteins and belong to the pre protein translocase pathway, were highly suppressed at 4 and 10 °C and during the 240 min exposure to 4 °C (T_5_), but they were shown to increase during transient cold acclimation (Additional files 5 and 6). Pre proteins bind to translocase and trigger cycles of ATP binding and hydrolysis so that pre protein substrates are transported at the expense of energy [55]. Suppression of certain housekeeping genes and metabolic activity including secretion could be a general response but important strategy to provide tolerance under cold stress conditions [56]. Some general response mechanisms to low temperature include higher turnover of macromolecules, tighter maintenance of intercellular pH, greater osmotic regulation, and motility, cessation of biomass production, and reduction of the activation energy before a pre-exponential growth phase [24, 56]. A gene (MrN33-118) that encodes a potassium transporter (Fig. 4, group P; Additional file 6) was suppressed under constant cold treatments (GT_4_, GT_10_), implying inhibitory controls over transmembrane ion movement to possibly save energy during cold conditions.

### Formation of symbiotic nitrogen-fixing nodules during cold stress

It has been shown that ß-glucan is implicated in the attachment of the bacterial cells to plant roots for the initiation of the symbiotic interaction [57]. ß-glucan also has an important role in hypo-osmotic adaptation [58]. Interestingly, the ß-glucan-transporting ATPase-encoding transcript MrN33-42b04 (Fig. 4, group Q; Additional file 6) was only induced (1.55 FC) during cold acclimation at condition GT_10_. As Arctic strains are more competitive than temperate strains in forming nodules at 10 °C in symbiosis with the temperate legume sainfoin (*Onobrychis vicifolia*) [59], our results suggest that the ß-glucan-transporting ATPase may play a crucial role in initiating symbiosis at low temperatures.

## Conclusions

The first partial transcriptome analysis of Arctic *Mesorhizobium* strain N33 under three different growing temperatures (4 °C, 10 °C and 21 °C), and cells exposed to a suboptimal temperature (4 °C) over a time course (2, 4, 8, 60 and 240 min) not only illustrated gene expression trends during cold treatment but also revealed some key molecular responses to low temperatures (Fig. 4). This experimental design was used to study the strain N33 transcriptome exposed to sub optimal temperatures because: i) strain N33 is able to nodulate and fix nitrogen at 10 °C while temperate rhizobia are inactive, and ii) this bacterium can still grow and survive at 4 °C albeit at a lower rate [60]. We were also interested in looking at gene expression changes after short exposures to cold (4 °C). We used 5760 genomic clones on microarray slides for gene expression analysis and focused on expression data for 134 genes significantly differentially expressed across treatments. This technological approach was taken since there was no genome sequence available for strain N33.

The low temperature transcriptome of strain N33 has revealed complex changes associated with cold exposure adaptation and constant growth at low temperature. The comparison of genomic, transcriptomic and proteomic analysis of other psychrophillic prokaryotes confirmed that cold adaptation is the result of multiple molecular changes in the cells [7, 13]. The greatest number of transcriptome changes in N33 during cold exposure was observed in genes involved in energy production and conversion, amino acid and carbohydrate transport and metabolism, translation, transcription, cell motility and secretion, cell envelope, and outer membrane biogenesis. Some of these changes have been observed in previous work on low temperature in other bacteria [8, 50]. Many of the transcripts identified encode proteins of unknown function, indicating our lack of knowledge of cold adaptation in prokaryotes. Further investigation of these genes will expand our insight into molecular traits for low temperature adaptation.

We observed that some genes that were significantly down-regulated are involved in housekeeping activities (e.g. metabolism, secretion, energy production, cell envelope and outer membrane biogenesis). Suppression of certain housekeeping genes at constant low temperature can reduce cell metabolism, conserve energy, and can be considered as an important tolerance strategy during cold stress. These results indicate that the physiological states of *Mesorhizobium* strain N33 are very different between short term and steady state cold exposure. Many more transcripts were significantly up or down regulated during the constant low temperature growth conditions compared to short term cold exposure.

Validation of candidate gene function (Fig. 5) using reverse genetics and site directed mutagenesis [43] is of interest in expanding our insights into molecular traits for low temperature adaptation in *Mesorhizobium* strain N33. The in-depth characterization of the transcriptomes using RNA-seq and whole genome sequence analysis of N33, currently underway, is the next step to identifying new cold adapted genes in strain N33. Furthermore, combining our metabolomics analysis [24] and future proteomics work will provide a system biology approach to understand cold adaptation in Arctic *Mesorhizobium* strain N33 to enhance nitrogen-fixing capacity and improve other agricultural traits in Canadian and other northern climates.

## Methods

### Bacterial strains, media cultures and growth conditions

The *Mesorhizobium* sp. strain N33 used in this study was previously isolated from Arctic legume *Oxytropis arctobia* [26]. Frozen glycerol stocks of strain N33 were used to inoculate 20 ml yeast mannitol broth (YMB) medium containing 200 μg ml^−1^ streptomycin [61] and incubated at 21 °C for 5 days on a rotary shaker (180 rpm). The purity of colonies was checked by cultivation on yeast extract mannitol agar (YMA) supplemented with 200 μg ml^−1^ streptomycin and 25 μg ml^−1^ Congo red [62]. Subsequent purity tests of the strain were conducted by performing a nodulation test on *Onobrychis viciifolia* (sainfoin), and by 16S-rDNA sequencing [63]. The sequence has been deposited in the NCBI nucleotide data base under accession number JX470579.

### Construction of genomic library and generation of target clones for microarray

*Mesorhizobium* strain N33 cells were grown in 100 ml of YMB media at 150 rpm at room temperature (~21 °C) to mid-log phase (OD_600_ = 0.4-0.6). The cells were precipitated, washed in TES buffer [64], and centrifuged (10,000 g) at 4 °C. Whole genomic DNA isolation was performed as described in 1989 by Laberge et al. [65] and DNA stored at −20 °C. DNA quality was verified using a NanoDrop 1000 spectrophotometer and by agarose gel (1 %) electrophoresis. Two μg of DNA was mechanically sheared using a nebulizer (Unomedical INC, McAllen, TX) under a nitrogen pressure of 20 psi for 3 min. DNA fragments ranging from 0.3–2 kb were subsequently blunt-ended using T4 DNA polymerase and Klenow DNA polymerase (Lucigen, Mississauga, ON), ligated into a pSMART-HC Kan vector (Lucigen), and transformed into electro-competent *E. coli* DH5α cells (Bio-Rad, Mississaugua, ON). Plasmid inserts were amplified by standard PCR from 5760 clones using the universal forward (SL1 = 5’-CAGTCCAGTTACGCTGGAGTC-3’) and reverse primers (SR2 = 5’-GGTCAGGTATGATTTAAATGGTCAGT-3’) complementary to vector sequences flanking the DNA inserts. Clones were digested with *Eco*RI (Boehringer Mannheim, Laval, QC) and fragment size and purity were evaluated by agarose gel electrophoresis. Some inserts were sequenced to verify that they were bona fide *Rhizobium* sequences by blast analysis with the GenBank database.

### Microarray printing

PCR reactions from the *Mesorhizobium* strain N33 library were purified using the QIAquick 96 PCR Purification Kit (Qiagen, Mississaugua, ON) and transferred into the 384 master plate format using a Multiprobe II EX Robotic Liquid Handling System (Perkin Elmer, Shelton, CT). Plates were then dried without heat in a DNA 120 Speed Vac (Savant Instruments Inc., Holbrook, NY). Dried samples were resuspended in 10 μl of 3XSSC and stored at −80 °C. A total of 13,056 features, consisting of duplicate spots for each clone and 32 spike-in and negative controls for each block were arranged and printed on Corning GAPS II coated slides, as per Corning instructions. Arrays were printed using the BioRobotics MicroGrid Compact spotter (Digilab Inc, Holliston, MA). The arrays were snap-dried and DNA UV cross-linked to the slides using a CL-1000 ultraviolet cross linker (UPV, Upland, CA) for a total of 300 mJ.

### RNA purification

A total of 8 conditions with 3 biological replicates, previously used for a metabolomic analyses of N33 [24], were screened in this experiment. N33 cells were grown in YMB medium to mid-log phase under three steady state growing temperature conditions (21 °C = control, GT_21_; GT_4_ = 4 °C, and GT_10_ = 10 °C). For the cold stress treatments, mid-log phase cells grown at 21 °C (T0) were exposed to 4 °C for different time points (T_1_ = 2 min, T_2_ = 4 min, T_3_ = 8 min, T_4_ = 60 min, T_5_ = 240 min in a rotary shaker water bath (150 rpm). After each treatment and before harvest of cells, transcription activity was halted by adding a stop solution [66]. The samples were immediately centrifuged (10,000 g) at 4 °C for 5 min. The pellets were washed once with cold TES buffer (100 mM NaCl, 1 mM EDTA, 10 mM Tris hydrochloride [pH 7.4]), re-centrifuged, and cells frozen in liquid nitrogen and stored at −85 °C. Total RNA was purified using the Epicenter Kit according to the manufacturer’s protocol with additional modifications: i) Small RNAs, proteins, carbohydrates, and DNA were precipitated with 5 M LiCl (Ambion). ii) DNA was also removed from RNA samples using DNase I (Ambion). Lack of DNA contamination of RNA samples was verified by standard PCR using one pair of primers from the OrfZ gene [28] of strain N33. RNA concentration was determined using the NanoDrop ND-1000 Spectrophotometer at 260 nm. RNA purity and integrity were assessed [64] by electrophoresis on a density gradient 1 % agarose/formaldehyde gel and using the Experion automated electrophoresis system (Bio-Rad).

### cDNA synthesis and labelling

cDNA synthesis, labelling, purification and hybridization were performed based on a protocol from The Institute for Genomic Research (TIGR, now the J. Craig Venter Institute) (ftp://ftp.jcvi.org/pub/data/PFGRC/MAIN/pdf_files/protocols/M007.pdf). Labelled probes were quantified on the NanoDrop ND-1000 Spectrophotometer and yields were calculated in picomoles of cDNA synthesized and Cy-dye incorporation. The labelled cDNA was dried in a vacuum centrifuge with medium heat for 45–60 min and stored at 4 °C in the dark. Human clones (~300 bp in length), purchased from Michigan State University were used as internal spike-in controls for hybridization.

### Microarray procedures

Two-colour hybridizations were performed using the samples from all cold treatments as “test” samples and the N33 room temperature sample as the “reference”. Dye-flip hybridizations were performed for each sample combination with three biological replicates for a total of 42 hybridizations. The labelled probes were combined with a solution containing 2 μl of calf thymus ss-DNA (10 mg/ml, Sigma), 2 μl Yeast tRNA (10 mg/ml, Invitrogen) and 61 μl of DIG easy hybridization buffer (Roche), denatured at 70 °C for 2 min and hybridized with the arrays at 37 °C for 8–18 h. Following incubation, the arrays were washed three times at 50 °C for 15 min in 1X SSC-0.1 % SDS, rinsed in 1x SSC and spun 5 min (1000 rpm) at room temperature. The slides were scanned sequentially at 532 and 635 nm using a Genepix 4200A microarray scanner (Molecular Devices, Sunnyvale, CA). Features were aligned in Genepix Pro 6.0 using the Irregular Features Spot Finder application and local background subtraction. Low quality features were flagged and filtered from further analysis. The raw data files were named using strain abbreviation, hybridization time date and slide number (i.e. MrN33-080326-13723296.gpr). The complete set of data for the N33 gene expression microarray experiment is available on the GEO (Gene Expression Omnibus) web site [67] under the accession number (GSE60710). http://www.ncbi.nlm.nih.gov/geo/query/acc.cgi?acc=GSE60710.

### Normalization and data analysis

Arrays were imported into Acuity 4 (Molecular Devices, Sunnyvale, CA Axon Instruments Inc.) for data normalization and analysis. The arrays were re-named by test treatment, test dye and biological replicate, respectively (i.e. GT_4__3_BR1). Lowess normalization was applied to each array [68, 69]. Data was filtered by removing features flagged in Genepix as low quality and removing spike-in controls and negative control features. Furthermore, we removed features with “Lowess A” intensities ≤8 in at least 32 of the arrays (75 %). The data type was changed to Lowess M log_2_ ratio. After the filtering steps, 4603 clones sequence remained in the dataset. Pearson centred hierarchical clustering analysis was carried out on the dataset and supported strong reproducibility of the technical and biological replicates across all treatments. Clustering was performed for data set over all treatments using self-organizing maps. To summarize the data set on an interval scale and show the variability of expression levels (log_2_ ratios) a box-whisker plot was created with the data using Genespring software (Agilent Technologies, Sunnyvale, CA, Version 11.5.1). The box-whisker plot [70] of expression levels represented the range of log_2_ ratios and a normal distribution over all the data range obtained in different cold treatments (data not shown). To narrow down the most differentially expressed clones based on a pattern of changes among different hypothermic conditions, a one-way analysis of variance (ANOVA) was performed on the Lowess M Log_2_ ratio data. Clones identified using ANOVA at p ≤ 0.005 (424 clones) were designated as differentially regulated datasets. The lists and names of 424 clones and their statistical p-value are available in Additional file 2. The false positive rates for the filtered transcripts were calculated (23 false positives expected for 424 clones at p ≤ 0.005) [71], and clustering was performed based on the individual SOM clusters and using Euclidean squared distances. In order to determine which of the 424 clones (p ≤ 0.005) are changing across all seven hypothermic conditions, an additional clustering using principal component analysis (PCA) [72] was performed (data not shown). We followed the criteria that are described in MIAME [73] for the microarray experiment and data analysis.

### Quantitative PCR

To validate the microarray results, qRT-PCR was carried out for 54 genes over 24 sscDNAs samples previously used for microarray hybridization. The Primer3-plus software [74] was used to design primers to recognize the specific gene within each genomic clone. The primer pairs used in qRT-PCR for each clone is shown in Additional file 5. Each sscDNA sample was synthesized from 10 μg of purified total RNA sample (as described previously) using Superscript III reverse transcriptase (Invitrogen) and Random primer Hexamers (Promega) at a final concentration of 25 ng/μl [66] with minor optimizations. The single-strand cDNAs were purified using QIAquick (Qiagen) PCR purification columns. The continuous fluorescent signals (SYBR Green I kit, Qiagen, Germany) detection was performed in a total of 35 cycles using the Applied Biosystem 7900HT Fast Real-Time PCR device in 384-well microplates. Each well contained 10 ng of sscDNA and a primer set (0.4 μM) in a 10 μl reaction mix. The specificity of primer sets were determined by dissociation curves and agarose gel electrophoresis. The qRT-PCR products were verified by randomly sequencing 30 PCR amplicons. The three most stably expressed genes were identified using gNorm algorithm and used as internal controls [31]. Quantification of the relative gene expression changes of the genes of interest were identified using the Cq (2^-ΔΔCT^) method [75] by comparison with the reference genes. The REST 2008 software [33] was used to verify the significant levels (p-values) of the gene expression. The experimental details on qRT-PCR analysis were based on the MIQE guidelines [32].

### Sequencing, bioinformatics, functional annotations and classification

The 424 differentially expressed clones (p < 0.005) were sequenced, assembled into 134 clones /contigs using SeqMan software (Lasergene, Inc.) (after discarding chimeric genomic clones) and annotated through NCBI GenBank, UniProtKB, KEGG, COG, and GO (Gene Ontology) molecular function via the QuickGO browser version 59 [76]. To decrease the number of sequences coding for hypothetical proteins, a literature-based GO annotation screen was applied, and all generated annotations were manually curated and reviewed. To assign transcripts into orthologous functional groups (COGs) and metabolic pathways [77], the differentially expressed sequences were translated into peptide sequences which were used to search in the COGnitor browser [78]. The COG database can now be searched using RPS-BLAST through the Conserved Domain Database web site, http://www.ncbi.nlm.nih.gov/Structure/cdd/wrpsb.cgi. We clustered the orthologus groups of proteins into higher and more detailed levels of functional groups using the COGs database (Additional file 3). Biochemical pathways were determined using COG and KEGG databases (Additional files 3 and 4). Clones/contigs were filtered and sorted based on the expression ratio (p < 0.005, ≥1.5 FC). The GC content of DNA sequences was calculated using the online tool http://www.sciencelauncher.com/oligocalc.html. The complete list of the genomic clones/contigs and their annotation is provided in Additional file 3. The consensus sequences for 134 clones/contigs were deposited in GenBank (accession numbers JX668791- JX668986, KR133469-KR133484).

### Availability of supporting data

The dataset supporting the results of this article is available in the NCBI GEO repository under accession GSE60710.

The consensus sequences for 134 genomic clones/contigs were deposited in NCBI GenBank (accession numbers JX668791 - JX668986, KR133469 - KR133484). All other supporting data are included within the article and its additional files.

## Additional files

Additional file 1: **Amplifying genomic clones of the Arctic** ***Mesorhizobium*** **strain N33 for array printing.** The genomic clone inserts were amplified by PCR using pSmart-SL1 (forward) and pSmart-SR2 (reverse) primers. The size of each insert was verified and applied as probes for DNA array experiments. In lane 1 the DNA ladder 1Kb (0.5 μg) was loaded. Lanes 2 to 17 show representative examples of the 5760 amplified N33clone inserts. All PCR products were assessed on 1 % agarose gel.

Additional file 2: **Expression values (log2 ratios) of 424 clones that were clustered using self-organization maps (SOM) with a statistical p value <0.005.**

Additional file 3: **Grouping the genes represented by 134 genomic clones/contigs into 3 clusters.** The genes were grouped into 3 clusters (43, 28, and 63) by self-organizing map (SOM). Sequences were analyzed by BLASTX in GenBank and or UniProtKB databases to identify related proteins. Functional annotation of the genes was performed using GO, COG, and KEGG databases.

Additional file 4: **List and description of the 73 genomic clones containing genes which displayed a minimum threshold of ± 1.5 fold change (p < 0.005) between treatments.**

Additional file 5: **Comparison of gene expression assay of qRT-PCR and microarray data in Arctic strain N33, and the primers used in this experiment.** To validate the microarray results, qRT-PCR was performed using the same cDNAs pool that was used to hybridize the microarrays. The array expression data is shown based on fold change ratio from a single representative genome clone. The complete list of specific primer pairs are provided for 54 selected clones that were used in qRT-PCR to validate the gene expression microarray data under hypothermic conditions in Arctic *Mesorhizobium* strain N33.

Additional file 6: **Description of the selected 52 genes that were differentially expressed in at least one data set (p < 0.005, ≥ ± 1.5 FC), which were used to create the heat maps (Fig.** **4** **).** The array expression data is the mean fold change ratio from all genomic clones belonging to a single contig.

## Abbreviations

CSPs: Cold shock proteins
CAPs: Cold acclimation proteins
MCPs: Methyl-accepting chemotaxis proteins
IBPs: Ice binding proteins
AFPs: Antifreeze proteins
ANOVA: Analysis of variance
SOM: Self-organizing maps
COGs: Clustering of the orthologous groups of proteins
NA: Not available
GO annotation: Gene ontology annotation
FC: Fold change
EPS: Exopolysaccharide
NADH: Nicotinamide adenine dinucleotide dehydrogenase
PMBP: Periplasmic mannitol-binding protein
ROS: Defence against reactive oxygen species
KEGG: Kyoto encyclopedia of genes and genomes
PCR: Polymerase chain reaction
qRT-PCR: Quantitative real-time PCR
PCA: Principal component analysis
MCSP: Major cold shock proteins
RNA-seq: RNA sequencing
YMA: Yeast extract mannitol agar
BLAST: Basic local alignment search tool
TES: Tris-EDTA solution
LiCl: Lithium chloride
cDNA: Complementary DNA
TIGR: The Institute for Genomic Research
SDS: Sodium dodecyl sulfate
GEO: Gene Expression Omnibus
LOWESS: Locally-weighted-regression
MIAME: minimum information about a microarray experiment
sscDNAs: Single strand cDNA
Cq: Quantification cycle in RT-PCR
REST: Relative expression software tool
MIQE: Minimum information for publication of quantitative real-time PCR experiments
AAFC: Agriculture and Agri-Food Canada
