# Supplementary figures and images for: Microarray transcriptional profiling of Arctic Mesorhizobium strain N33 at low temperature provides insights into cold adaption strategies
Source: BMC Genomics. 2015 May 15;16(1):383. doi: 10.1186/s12864-015-1611-4 (PMC4432818; doi:10.1186/s12864-015-1611-4)

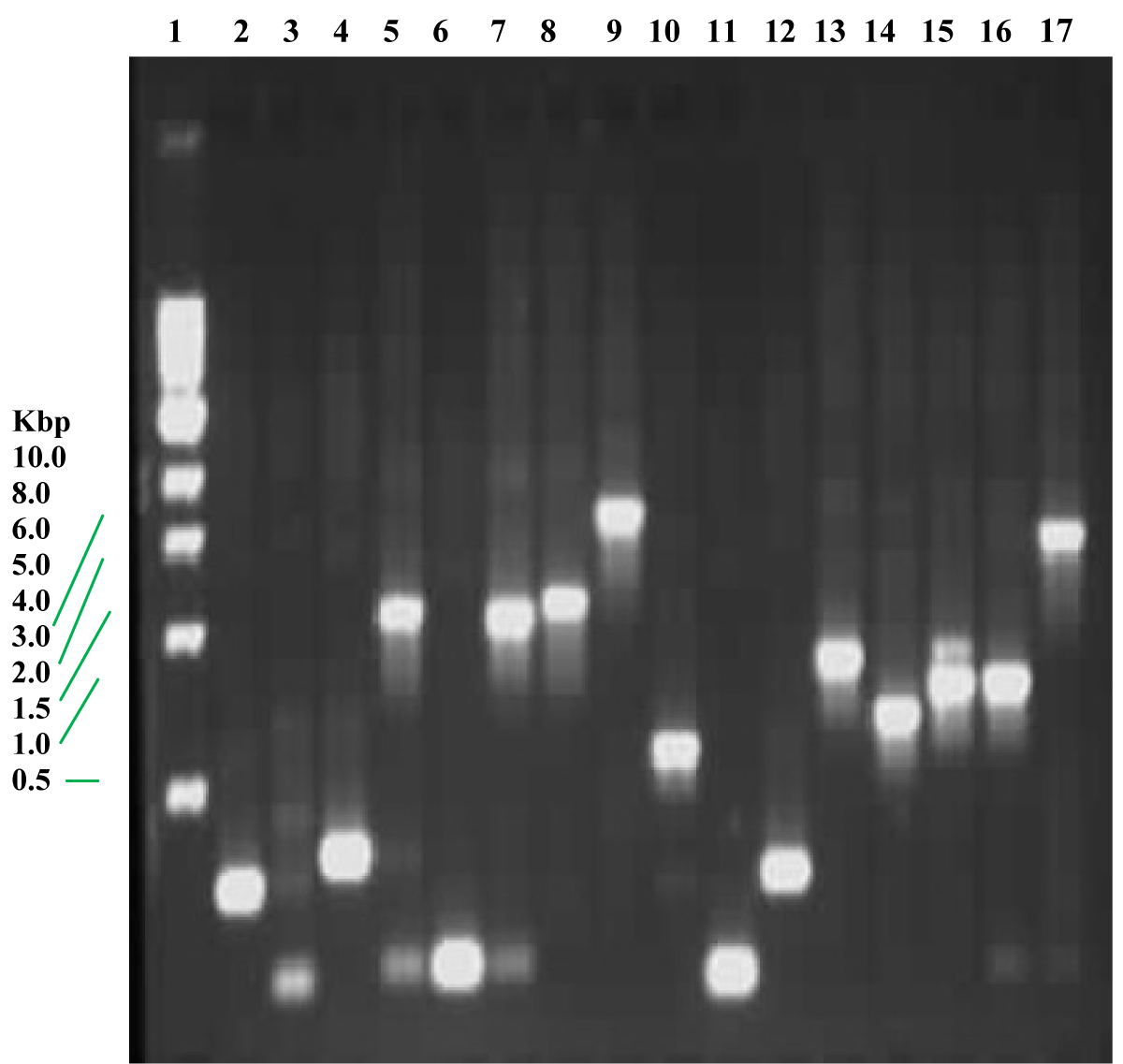

Supplement: Supplementary file 1 — Amplifying genomic clones of the Arctic Mesorhizobium strain N33 for array printing. The genomic clone inserts were amplified by PCR using pSmart-SL1 (forward) and pSmart-SR2 (reverse) primers. The size of each insert was verified and applied as probes for DNA array experiments. In lane 1 the DNA ladder 1Kb (0.5 μg) was loaded. Lanes 2 to 17 show representative examples of the 5760 amplified N33clone inserts. All PCR products were assessed on 1 % agarose gel. [file 12864_2015_1611_MOESM1_ESM.tiff]
